# Cannabidiol-Dominant *Cannabis sativa* L. Inflorescence Extract Ameliorates Atopic Dermatitis by Modulating NLRP3 Inflammasome and JAK1/STAT6 Signaling in DNCB-Induced Mice

**DOI:** 10.3390/nu18142382

**Published:** 2026-07-21

**Authors:** Ji-Ye Han, Do-Won Lim, Osoung Kwon, Yun Jung Lee, Minji Choi, Bori Lee, Soohyang Noh, Mansoo Cho, Young-Mi Lee

**Affiliations:** 1 Department of Oriental Pharmacy, College of Pharmacy, and Wonkwang-Oriental Medicines Research Institute, Wonkwang University, Iksan 54538, Republic of Korea; hsue0112@gmail.com (J.-Y.H.); shrons@wku.ac.kr (Y.J.L.);; 2 The One Health Design, Iksan 54575, Republic of Korea; 3 Graduate School of Techno Design, Kookmin University, Seoul 02707, Republic of Korea

**Keywords:** *Cannabis sativa* L., atopic dermatitis, cannabinoid, nutraceuticals, NLRP3 inflammasome

## Abstract

Background/Objectives: Atopic dermatitis (AD) is a chronic inflammatory skin disorder requiring sustainable therapeutic alternatives. *Cannabis sativa* L. is a valuable industrial crop rich in bioactive secondary metabolites; its potential as a standardized functional ingredient for promoting skin health has not yet been fully investigated. This study aimed to evaluate the therapeutic effects of a chemically characterized *C. sativa* inflorescence ethanol extract (CSE) on AD. Methods: To evaluate the efficacy of CSE, phytochemical profiling was performed using UPLC, and its underlying molecular mechanisms were investigated in a DNCB-induced mouse model. Results: UPLC analysis was employed to establish the phytochemical profile, identifying 15 cannabinoids and quantifying 8 major components. CBDA was the most abundant component, with a content of 261.79 mg/g in the extract. In a DNCB-induced mouse model, CSE significantly reduced mast cell infiltration and serum IgE levels while downregulating Th2-associated cytokines. At the molecular level, CSE inhibited the activation of the MAPK, NLRP3 inflammasome, and JAK1/STAT6 signaling pathways. Crucially, CSE treatment substantially increased the expression of skin barrier proteins, such as filaggrin and involucrin, thereby enhancing skin hydration. Conclusions: These findings suggest CSE as a high-value functional ingredient capable of ameliorating AD by modulating multi-target immune responses. This study provides a robust scientific basis for utilizing standardized *C. sativa* inflorescence as a potent functional ingredient or a nutraceutical agent for the management of chronic skin inflammatory conditions.

## 1. Introduction

*Cannabis sativa* L., a prominent member of the Cannabaceae family, has been recognized for centuries as a versatile and high-value agricultural crop with extensive applications in fiber production and medicinal use. Originating in Central Asia, this species has been widely disseminated and is now cultivated across diverse geographical regions as a sustainable bioresource. The phytochemical diversity of *C. sativa*, primarily characterized by the concentration of tetrahydrocannabinol (THC), dictates its industrial utility; while low-THC cultivars are favored for agricultural and food production, high-THC varieties are predominantly utilized in pharmaceuticals [1]. Specifically for medical applications, cultivation conditions significantly influence the concentrations of key bioactive compounds, such as cannabinoids and terpenoids [2]. This variability leads to diverse chemical profiles [3]. Currently, *C. sativa* is utilized across various sectors, ranging from construction materials, textiles, food ingredients, and medical applications [2].

Beyond its industrial versatility, *C. sativa* serves as a rich natural reservoir of secondary metabolites, exhibiting a wide range of therapeutic effects, including antioxidant, anti-inflammatory, and analgesic activities [4]. These biological properties are largely attributed to its unique phytochemical profile, which comprises over 1000 bioactive compounds [5]. While phytocannabinoids are the most prominent constituents, the plant also contains significant amounts of terpenoids and flavonoids, which contribute to its overall pharmacological synergy [6]. To date, approximately 125 phytocannabinoids have been structurally elucidated [7]. Although THC and cannabidiol (CBD) remain the most extensively studied for their clinical applications, there is a growing paradigm shift in food chemistry toward minor cannabinoids such as cannabigerol (CBG), cannabichromene (CBC), and cannabinol (CBN) [8]. These minor components are garnering significant scientific interest due to their distinct pharmacological properties, suggesting that the comprehensive phytochemical standardization of *C. sativa* inflorescences is crucial for their successful application in the nutraceutical and functional food industries.

AD is a chronic, relapsing inflammatory skin disease characterized by pruritus, eczematous lesions, and skin barrier dysfunction. It is considered a refractory condition due to its tendency for frequent exacerbations and limited long-term treatment response. Although AD is a common skin disorder in pediatric populations, it remains a challenging condition to fully cure [9]. AD is a multifactorial disease in which genetic, immunological, and environmental factors contribute to its pathogenesis through complex and interrelated mechanisms. Among these, T helper type 2 (Th2) immune responses and skin barrier function play critical roles, and abnormalities in these mechanisms exacerbate skin inflammation and induce severe pruritus [10].

Th2 cytokines, particularly interleukin (IL)-4 and IL-13, serve as pivotal mediators in the pathogenesis of AD. These cytokines drive B cell class switching toward immunoglobulin E (IgE) production and promote eosinophilic inflammation [11]. In addition to amplifying allergic responses, IL-4 and IL-13 disrupt epidermal barrier integrity by downregulating filaggrin and other barrier-associated proteins, thereby perpetuating chronic inflammation and disease severity.

Epidermal barrier abnormalities, including functional defects in filaggrin, are commonly observed pathological features of AD lesions. Defects in filaggrin expression, resulting from genetic mutations or the effects of Th2 cytokines, compromise barrier integrity, leading to increased transepidermal water loss, heightened susceptibility to infection, and subsequent skin dryness and pruritus [12]. Given the chronic and relapsing nature of these barrier defects and immune dysregulation, the utilization of plant-derived bioactive compounds represents a promising strategy for long-term management, owing to their potential for multi-target efficacy and safety.

Previous studies have reported that CBD reduces edema and suppresses inflammation in in vivo models, thereby demonstrating therapeutic effects in AD [13]. Despite these promising findings, the complex synergistic effects of the diverse phytochemicals present in *C. sativa* extracts remain largely unexplored, and the precise molecular mechanisms underlying their action on the skin barrier and immune signaling are not yet fully elucidated. Since the biological efficacy of plant extracts is intrinsically linked to their phytochemical standardization, a comprehensive investigation into the multi-target effects of whole-plant extracts is essential for their development as reliable therapeutic agents.

In this context, this study aimed to evaluate the therapeutic efficacy of a chemically characterized 70% ethanol extract of *C. sativa* inflorescence—a variety developed in the Republic of Korea—on 2,4-Dinitrochlorobenzene (DNCB)-induced AD in mice. Furthermore, the underlying chemical-biological interactions were elucidated by analyzing the modulation of the MAPK, NLRP3 inflammasome, and JAK1/STAT6 signaling pathways, alongside the restoration of key skin barrier proteins.

## 2. Materials and Methods

### 2.1. Preparation of C. sativa Inflorescence Ethanol Extract (CSE)

In this study, *C. sativa* strain No. IT342820, sourced from the National Agrobiodiversity Center (RDA, Jeonju, Republic of Korea), was utilized as the experimental material. The *C. sativa* inflorescences were provided by Nongboomind (Andong, Republic of Korea). Immediately after harvest, the inflorescences were subjected to hot-air drying at 50 °C for 24 h. Subsequently, the dried samples were vacuum-packed and stored in the dark at a controlled room temperature (20–25 °C) until extraction. The dried *C. sativa* inflorescences (10 g) were subjected to three repeated extractions with 150 mL of 70% ethanol by ultrasonic irradiation (40 kHz, 40–50 °C) for 30 min each. After filtration through a 6 μm filter paper (ADVANTEC, Tokyo, Japan), the solution was concentrated using a rotary vacuum evaporator (EYELA N-1110, Tokyo, Japan) at 40 °C under reduced pressure. Following lyophilization, a powder (CSE) was obtained with a final yield of 28.26%. CSE was stored at −20 °C until use.

### 2.2. Comprehensive Phytochemical Profiling and Quantitative Analysis of Cannabinoids in CSE

The phytochemical profiling of CSE was performed using Ultra-Performance Liquid Chromatography (UPLC). The phytochemical constituents of CSE were analyzed using a Waters ACQUITY UPLC H-Class system (Waters, Milford, MA, USA) equipped with a photodiode array (PDA) detector. For the identification and quantification of cannabinoids, certified reference standards were purchased from multiple sources: a cannabinoid mixture and individual standards were obtained from Cayman Chemical (Ann Arbor, MI, USA) and Absolute Standards, Inc. (Hamden, CT, USA). Additionally, high-purity reference standards for major cannabinoids were purchased from the U.S. Pharmacopeia (USP; Rockville, MD, USA). The cannabinoid standards were dissolved in acetonitrile to a final concentration of 1 mg/mL and used for the analysis. Chromatographic separation was performed on an ACQUITY UPLC BEH C18 column (130 A, 1.7 um, 2.1 mm × 100 mm) coupled with an ACQUITY UPLC BEH C18 VanGuard pre-column (130 A, 1.7 um, 2.1 mm × 5 mm). The mobile phase consisted of 0.1% formic acid in water (solvent A) and 0.1% formic acid in a mixture of acetonitrile and methanol (25:75, *v*/*v*; solvent B). The gradient elution program was optimized as follows: 0–1 min, 73.5% B; 1–5 min, 73.5–77.0% B; 5–9 min, 77.0–90.0% B; 9–11 min, 90.0% B; 11–11.1 min, 90.0–73.5% B; and 11.1–15 min, 73.5% B for re-equilibration. The flow rate was maintained at 0.35 mL/min, and the injection volume was 1.5 μL. The column temperature was kept constant at 30 °C. The PDA detector scanned a range of 190 to 400 nm, and the target cannabinoids were monitored and quantified at a detection wavelength of 220 nm.

### 2.3. Experimental Animals

Six-week-old male BALB/c mice were purchased from SAMTAKO BIO REPUBLIC OF KOREA (Osan, Republic of Korea). All animals were housed under a 12 h light/dark cycle in a room maintained at 24 ± 1 °C and 50 ± 5% humidity, with *ad libitum* access to food and water. This study was conducted in accordance with the ethical guidelines approved by the Wonkwang University Animal Experiment Ethics Committee (Confirmation No. WKU24-76, dated 17 December 2024).

### 2.4. Induction of AD-like Skin Lesions in Mice

The induction of AD-like lesions was performed using a protocol based on the method by Riedl et al., with some minor modifications [14]. The doses of CSE (100 and 200 mg/kg) and dexamethasone (1 mg/kg) were determined based on body weight, corresponding to CSE concentrations of 2% and 4% (*w*/*v*) in a 100 μL volume. These dosages were selected in consideration of the experimental period and findings from previous studies [15]. Mice were housed for one week to acclimate to their new environment. Following the acclimation period, the dorsal hair was removed using a razor while the mice were under anesthesia with isoflurane (Hana Pharm, Seoul, Republic of Korea). Mice were randomly divided into five groups (*n* = 5 per group): (1) Normal group with vehicle treatment, (2) DNCB-sensitized control group with vehicle treatment, (3) DNCB-sensitized group treated with dexamethasone (positive control), (4) DNCB-sensitized group treated with CSE 100 mg/kg, (5) DNCB-sensitized group treated with CSE 200 mg/kg. All samples were dissolved in corn oil before administration, and DNCB was dissolved in an acetone: olive oil (4:1, *v*/*v*). After hair removal, the mice underwent a two-stage DNCB application. Mice were sensitized with 1% DNCB (4 times in Week 1), followed by a challenge with 0.5% DNCB (3 times/week for 2 weeks) to induce AD-like lesions. CSE and dexamethasone were orally administered every two days for a total of three weeks.

### 2.5. Evaluation of Clinical Score

A standard dermatitis score was utilized to assess the severity of AD-like skin lesions [16]. A cumulative score for dermatitis severity was calculated by individually scoring four clinical signs—erythema, dryness, scarring/abrasions, and swelling—on a scale of 0 to 3. Dermatitis scores were evaluated weekly by two independent researchers blinded to the treatment groups.

### 2.6. Histological Analysis

At the end of the experiment, dorsal skin tissues were collected and fixed in 10% neutral buffered formalin (NBF). The tissues were then dehydrated through a graded ethanol series, cleared in xylene, and embedded in paraffin. Paraffin-embedded tissues were serially sectioned at 4 µm. The sections were deparaffinized in xylene, rehydrated through a graded ethanol series, and stained with hematoxylin and eosin (H&E) or toluidine blue. Micrographs were acquired using an EasyScan Pro 6 digital slide scanner (Motic, Xiamen, China), and epidermal thickness and mast cell infiltration were evaluated in three randomly selected sections per sample.

### 2.7. Quantitative Polymerase Chain Reaction (qPCR)

Total RNA was isolated from tissues using the RiboEx™ kit (GeneAll Biotechnology, Seoul, Republic of Korea). The isolated RNA was then reverse-transcribed into cDNA using the HelixCript™ Easy cDNA Synthesis kit (NanoHelix, Daejeon, Republic of Korea). For quantitative analysis, qPCR was performed on a StepOnePlus Real-Time PCR System (Applied Biosystems, Foster City, CA, USA) with the RealHelix Premier qPCR kit (NanoHelix, Daejeon, Republic of Korea). All procedures were carried out according to the manufacturers’ protocols. Primer sequences for gene amplification are provided in Table 1.

### 2.8. Western Blot Analysis

Proteins were extracted by homogenizing the tissue in radioimmunoprecipitation assay (RIPA) buffer (ELPIS-Bioech, Daejeon, Republic of Korea) containing a Halt™ protease inhibitor cocktail (Thermo Fisher Scientific, Cleveland, OH, USA). The concentration of the extracted proteins was determined using the Bradford assay. The samples were then separated by sodium dodecyl sulfate-polyacrylamide gel electrophoresis on a 10% polyacrylamide gel and transferred to a polyvinylidene fluoride membrane. The membrane was subsequently blocked in 5% skim milk (*w*/*v*) for 1 h at room temperature. After washing, the membrane was incubated with the primary antibody overnight at 4 °C, followed by incubation with the secondary antibody for 1 h at room temperature. Primary antibodies were used at the following concentrations: p-ERK (1:1000), ERK (1:1000), p-JNK (1:1000), JNK (1:1000), p-p38 (1:1000), p38 (1:1000), NLRP3 (1:500), caspase-1 (1:1000), IL-1β (1:1000), p-JAK1 (1:1000), JAK1 (1:1000), p-STAT6 (1:1000), STAT6 (1:1000), filaggrin (1:1000), involucrin (1:1000), β-actin (1:1000). Primary antibodies against filaggrin and involucrin were purchased from Thermo Fisher Scientific (Cleveland, OH, USA) and Santa Cruz Biotechnology, Inc. (Santa Cruz, CA, USA), respectively. All other primary antibodies were obtained from Cell Signaling Technology, Inc. (Danvers, MA, USA). Proteins were detected using an enhanced chemiluminescence reagent (Kindle Biosciences, Greenwich, CT, USA) and imaged with a ChemiDoc imaging system (Bio-Rad, Hercules, CA, USA).

### 2.9. Statistical Analysis

All data are presented as the mean ± standard deviation (SD) from at least three independent experiments. All groups were analyzed using one-way analysis of variance (ANOVA) followed by Tukey’s multiple comparison test. To ensure visual clarity, only representative significant differences are displayed in the figures. Detailed information regarding the specific groups compared is provided in each figure legend. Differences were considered statistically significant at * *p* < 0.05, ** *p* < 0.01, and *** *p* < 0.001. All statistical analyses were performed using GraphPad Prism version 8.0 (GraphPad Software, San Diego, CA, USA).

## 3. Results

### 3.1. Quantitative Determination and Phytochemical Profiling of Cannabinoids in CSE

**Figure 1:**
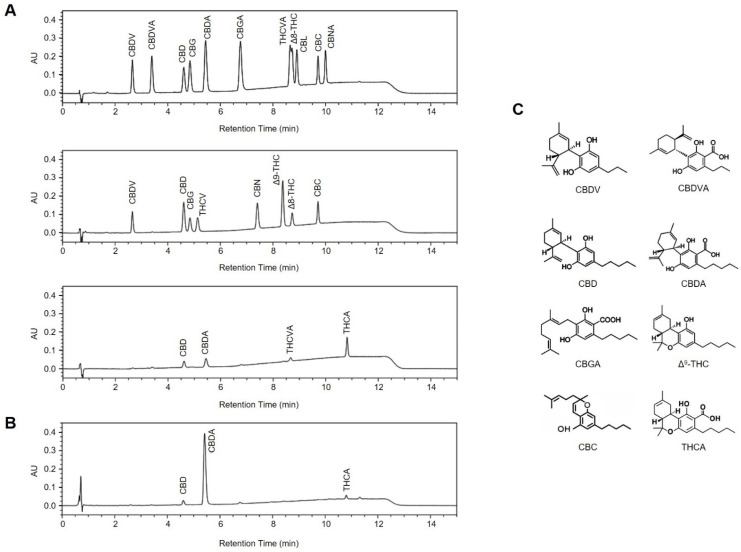
Representative UPLC chromatograms of cannabinoids in CSE. The peaks of identified cannabinoids are labeled with their respective abbreviations. Peak identification was confirmed by comparing retention times with reference standards. (**A**) Chromatogram of cannabinoid standards. The top panel shows the separation of an individual standard mixture; the middle panel displays the neutral form standard mixture; the bottom panel represents the acidic form standard mixture. (**B**) Chromatogram of CSE. (**C**) Chemical structures of cannabinoids detected in CSE. Abbreviations: CBDV, cannabidivarin; CBDVA, cannabidivarinic acid; CBD, cannabidiol; CBG, cannabigerol; THCV, tetrahydrocannabivarin; CBDA, cannabidiolic acid; CBGA, cannabigerolic acid; CBN, cannabinol; Δ^9^-THC, delta-9-tetrahydrocannabinol; THCVA, tetrahydrocannabivarinic acid; Δ^8^-THC, delta-8-tetrahydrocannabinol; CBL, cannabicyclol; CBC, cannabichromene; CBNA, cannabinolic acid; THCA, tetrahydrocannabinolic acid.

A profiling of 15 cannabinoids was performed via UPLC, and a total of eight cannabinoids were identified and quantified in the CSE (Figure 1). Among these, the combined content of cannabidiolic acid (CBDA) and CBD was 278.18 mg/g, which accounted for 27.81% of the total extract weight (Table 2).

### 3.2. CSE Ameliorates AD-like Symptoms in DNCB-Induced Mice

**Figure 2:**
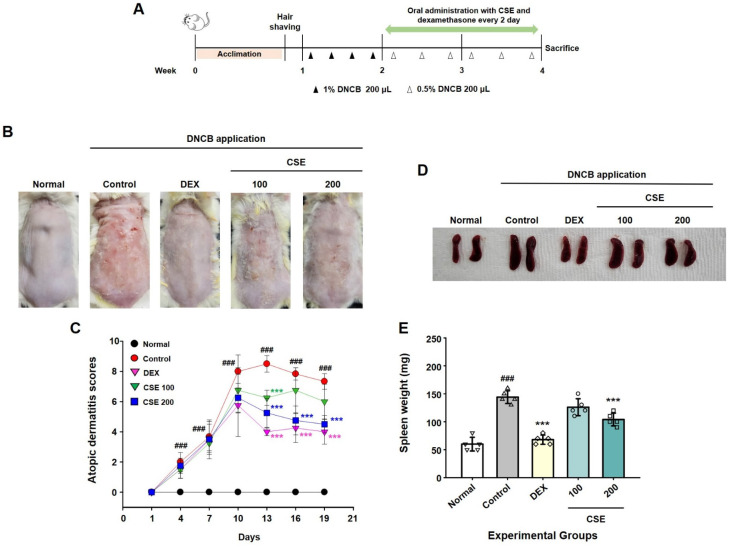
Ameliorative effect of CSE on AD symptoms in DNCB-induced mice. (**A**) Schematic diagram of AD induction in mice. An AD-like skin lesion model was established in BALB/c mice by repeated topical application of DNCB. Mice were divided into five groups: Normal (vehicle only), Control (DNCB only), DEX (DNCB + dexamethasone 1 mg/kg), CSE 100 (DNCB + CSE 100 mg/kg), and CSE 200 (DNCB + CSE 200 mg/kg). (**B**) Representative AD lesion images of dorsal skin lesions from each group. (**C**) Clinical severity scores based on four criteria: erythema, dryness, scarring/abrasions, and swelling. (**D**) Representative images of spleens from each group. (**E**) Spleen weights of mice in each group. Data presented as means ± SD (*n* = 5). ^###^ *p* < 0.001 vs. Normal group; *** *p* < 0.001 vs. Control group.

To establish an AD-like animal model, BALB/c mice were topically sensitized with DNCB for 3 weeks. Dexamethasone (positive control) and CSE were then administered orally every other day (Figure 2A). DNCB sensitization successfully induced AD-like symptoms on the dorsal skin, which were significantly alleviated by CSE (Figure 2B). AD symptoms in DNCB-induced mice became markedly significant compared to the normal group after 4 days. A notable difference was subsequently observed between the control and CSE-treated groups after 10 days (Figure 2C). In addition, DNCB-induced splenomegaly was markedly reduced by CSE treatment, with the high-dose group exhibiting a significant difference compared to the control group (Figure 2D,E).

### 3.3. CSE Reduces Both Epidermal and Dermal Thickening, as Well as Mast Cell Infiltration in Skin Lesions

**Figure 3:**
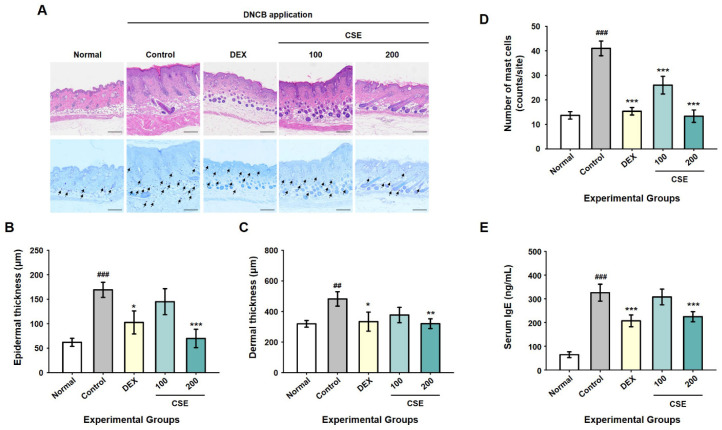
Effects of CSE on histopathological alterations in DNCB-induced Mice. Following H&E staining, the thicknesses of the epidermis and dermis were measured, and mast cell infiltration was assessed using toluidine blue staining. (**A**) Histological analysis of dorsal skin using H&E and toluidine blue staining (magnification 40×, scale bar = 400 μm, arrows indicate mast cells). (**B**) Epidermal thickness and (**C**) dermal thickness of dorsal skin. The thicknesses were measured using ImageJ 1.54 at three randomly selected points per section. (**D**) The number of infiltrated mast cells. The toluidine blue-positive cells were counted in three randomly selected sites (high-power fields) per section. (**E**) Serum IgE concentrations in each group. Data are presented as means ± SD (*n* = 3). ^##^ *p* < 0.01 and ^###^ *p* < 0.001 vs. Normal group; * *p* < 0.05, ** *p* < 0.01 and *** *p* < 0.001 vs. Control group.

Histological analysis using H&E staining demonstrated that CSE treatment markedly reduced the thickness of both the epidermis and dermis. Also, the high-dose CSE group exhibited a more pronounced reduction in epidermal thickness than that observed in the dexamethasone-treated group (Figure 3A–C). Toluidine blue staining indicated that CSE treatment significantly reduced mast cell infiltration by DNCB sensitization (Figure 3A,D). Serum IgE levels were considerably elevated by DNCB sensitization and were reduced following CSE treatment. High-dose CSE exhibited effects comparable to those of dexamethasone (Figure 3E).

### 3.4. CSE Suppresses Th2-Associated Cytokines in DNCB-Induced Mice

**Figure 4:**
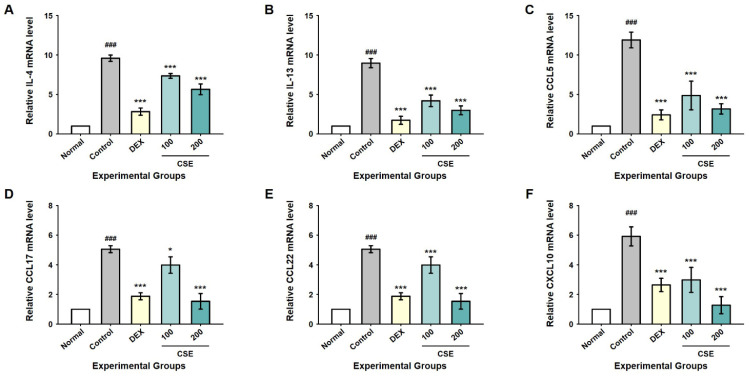
CSE-mediated suppression of Th2-associated cytokines and chemokines in DNCB-induced mice. Gene expression was analyzed by quantitative PCR using whole tissue lysates. The mRNA levels of (**A**) IL-4, (**B**) IL-13, (**C**) CCL5, (**D**) CCL17, (**E**) CCL22, and (**F**) CXCL10 were measured and normalized to GAPDH. Data are presented as means ± SD (*n* = 3). ^###^ *p* < 0.001 vs. Normal group; * *p* < 0.05 and *** *p* < 0.001 vs. Control group.

The gene expression of IL-4 and IL-13 was reduced in the CSE-treated group compared with the control group. Similarly, the expression of C-C motif chemokine ligand (CCL) 5, CCL17, CCL22, and C-X-C motif chemokine ligand (CXCL) 10 was significantly decreased, with the most pronounced reductions observed for CCL5 and CXCL10. Notably, the downregulation of CCL17, CCL22, and CXCL10 induced by CSE was greater than that induced by dexamethasone (Figure 4).

### 3.5. CSE Inhibits the Activation of MAPK and NLRP3 Inflammasome in DNCB-Induced Mice

**Figure 5:**
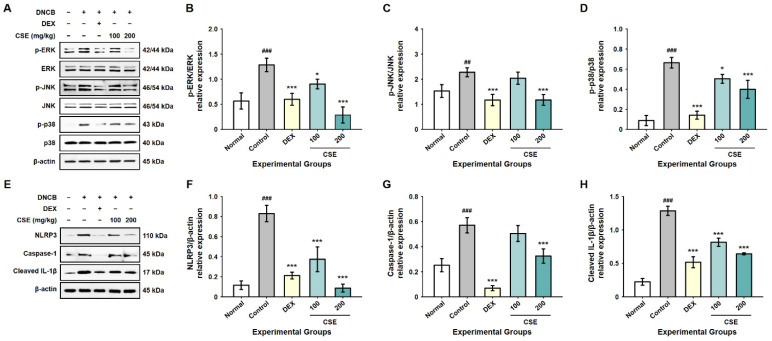
Inhibitory effects of CSE on MAPK and NLRP3 inflammasome signaling pathways in DNCB-induced mice. Protein expression was analyzed by Western blotting using whole tissue lysates. (**A**) Representative Western blot images showing both total and phosphorylated forms of ERK, JNK, and p38. Graphs represent the ratios of phosphorylated to total protein levels of (**B**) ERK, (**C**) JNK, and (**D**) p38. (**E**) Representative Western blot images showing NLRP3, caspase-1, and cleaved IL-1β. The graphs represent the ratios of (**F**) NLRP3, (**G**) caspase-1, and (**H**) cleaved IL-β protein levels to β-actin protein levels. Data are presented as means ± SD (*n* = 3). ^##^ *p* < 0.01 and ^###^ *p* < 0.001 vs. Normal group; * *p* < 0.05 and *** *p* < 0.001 vs. Control group.

The DNCB-induced phosphorylation of ERK, JNK, and p38 was distinctly attenuated by CSE treatment in dorsal skin. Among the phosphorylated MAPK proteins, the reduction in ERK was the most marked, with its levels decreased by approximately 3.0-fold, compared with 2.0- and 1.7-fold decreases observed in JNK and p38, respectively (Figure 5A–D). The NLRP3 inflammasome was inhibited by CSE treatment, as evidenced by the reduced levels of NLRP3 and caspase-1, along with decreased levels of cleaved IL-1β. Notably, the reduction in NLRP3 was the most significant of all the measured proteins (Figure 5E–H).

### 3.6. CSE Downregulates the JAK1/STAT6 Pathway and Increases Skin Barrier Factors

**Figure 6:**
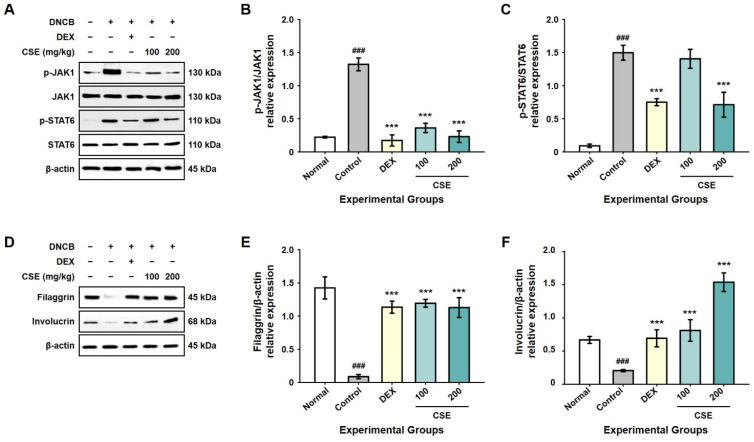
Inhibition of JAK1/STAT6 phosphorylation and restoration of skin barrier factors by CSE in DNCB-induced mice. Phosphorylation levels of JAK1 and STAT6, as well as the expression levels of skin barrier proteins, were evaluated by Western blotting. (**A**) Representative Western blot images of total and phosphorylated forms of JAK1 and STAT6. Quantification of the ratios of (**B**) phosphorylated JAK1 to total JAK1 and (**C**) phosphorylated STAT6 to total STAT6. (**D**) Representative Western blot images of filaggrin and involucrin. The graphs represent the ratios of (**E**) filaggrin and (**F**) involucrin protein levels to β-actin protein levels. Data are presented as means ± SD (*n* = 3). ^###^ *p* < 0.001 vs. Normal group; *** *p* < 0.001 vs. Control group.

The DNCB-induced phosphorylation of JAK1 was significantly attenuated by both CSE and dexamethasone treatment. Importantly, the levels of phosphorylated JAK1 were normalized to those of the normal control group in the high-dose CSE group. The DNCB-induced phosphorylation of STAT6 was effectively inhibited by dexamethasone and high-dose CSE treatment; however, no statistically significant effect was observed with the low-dose CSE group (Figure 6A–C). Conversely, filaggrin and involucrin, which are key skin barrier factors, were significantly suppressed by DNCB sensitization. Both filaggrin and involucrin were effectively restored by dexamethasone and CSE treatment; furthermore, involucrin was substantially elevated, with an increase of more than 2.0-fold in the high-dose CSE group (Figure 6D–F).

## 4. Discussion

The findings of this study establish CSE as a potent therapeutic agent for AD, exerting comprehensive inhibitory effects through its constituent cannabinoids on multi-target pathways that alleviate both clinical severity and underlying inflammatory responses at the molecular level.

The chemical distinction between industrial hemp and marijuana is primarily defined by the concentration of Δ^9^-THC, with a regulatory threshold of 0.3% (*w*/*w*) on a dry weight basis [1]. In this study, phytochemical analysis confirmed that the total THC content was 0.27% on a dry weight basis, effectively validating its classification as industrial hemp under international standards (Table 2). In contrast, the total CBD content was found to be 6.95% of the dry weight, indicating a high accumulation of non-psychoactive, therapeutic cannabinoids. This chemical profile—characterized by compliant THC levels and high CBD abundance—underscores the safety, legality, and pharmaceutical potential of CSE as a functional ingredient for AD treatment.

AD presents with core clinical features of pruritus and xerosis, often accompanied by acute inflammatory signs such as erythema and edema, as well as chronic features like lichenification [17]. The clinical symptoms of DNCB-induced AD-like lesions were improved by CSE administration. Specifically, similar effects were observed with high-dose CSE to those of dexamethasone, a steroid used for inflammatory diseases, from the third week onward (Figure 2B,C). According to previous studies, T cell activation-induced splenomegaly was observed in animal models of AD [18]. Splenomegaly was observed in the DNCB-induced group, while spleen size recovered in the CSE-treated group (Figure 2D,E). These results suggest that CSE alleviates the clinical symptoms of AD and suppresses excessive immune responses in DNCB-induced mice.

Hyperplasia of skin tissue is considered a major feature of the chronic AD stage, which is predominantly driven by increased inflammatory cell infiltration [19]. In AD lesions, an increase in mast cells is additionally observed, and mast cell degranulation contributes to the pruritus of AD [20]. Hyperplasia of the epidermis and dermis was induced by DNCB sensitization, and this effect was significantly reduced by CSE application. Furthermore, mast cell infiltration and serum IgE levels in the high-dose CSE group were observed to be similar to those in the dexamethasone-treated group (Figure 3). These findings indicate that CSE exerts an anti-inflammatory effect by inhibiting inflammatory cell infiltration in DNCB-induced AD-like lesions.

Consistent with the established role of Th1/Th2 imbalance in AD pathogenesis [21,22], our results showed that CSE application effectively modulated both Th1- and Th2-related inflammatory markers. Specifically, CSE treatment significantly downregulated Th2-associated cytokines (IL-4, IL-13) and chemokines (CCL5, CCL17, CCL22), which are pivotal in the acute phase of AD (Figure 4). Furthermore, the reduction in CXCL10 expression—a potent inducer of Th1 migration during the chronic phase [23]—suggests that CSE may exert therapeutic effects across both acute and chronic stages of AD. Notably, the high-dose CSE group exhibited a 2- to 4-fold decrease in these inflammatory mediators, indicating its robust potential to inhibit the migration and activation of key inflammatory cells.

CSE further exhibited anti-inflammatory efficacy by suppressing MAPK-mediated cytokine transcription and NLRP3 inflammasome activation (Figure 5). Furthermore, the reduced IL-1β secretion via NLRP3 inflammasome attenuation aligns with previous studies identifying this pathway as a key mediator of skin inflammation [24]. These results indicate that CSE effectively inhibits both the transcription and maturation phases of inflammatory cytokine production. Crucially, CSE potent inhibitory effects extended to the JAK1/STAT6 signaling, a primary mediator of IL-4 and IL-13 signaling [25,26]. In this study, CSE application significantly suppressed the phosphorylation of JAK1 and STAT6, showing efficacy comparable to dexamethasone (Figure 6A–C). This modulation is likely driven by the high concentration of CBD and minor cannabinoids like CBC, which are known to regulate JAK/STAT cascades through cannabinoid receptor interaction [27,28].

Beyond the modulation of inflammatory signaling, CSE treatment contributed to the restoration of skin barrier function. Unlike the characteristic barrier impairment and loss of filaggrin observed in AD [29], CSE significantly upregulated the expression of filaggrin and involucrin (Figure 6D–F). This improvement is consistent with previous studies on cannabinoid-induced barrier enhancement [30], further supporting the role of CSE in reinforcing the skin’s structural integrity through the upregulation of these key barrier factors. Collectively, these results demonstrate that CSE exerts a multi-targeted therapeutic effect by simultaneously suppressing intracellular inflammatory signaling and repairing the epidermal barrier.

CSE, which exhibits anti-atopic dermatitis effects, holds significant value as a functional ingredient. Since AD requires long-term management, CSE serves as a safe, plant-based nutritional intervention. It could effectively complement conventional therapies while minimizing the risk of steroid-related side effects. Furthermore, CSE has broad potential for application in the nutraceuticals and cosmeceutical industries, offering a high-value, versatile solution for both systemic and dermatological health.

Despite the significant findings, certain limitations exist in the present study. Although the major mechanisms were identified in a DNCB-induced mouse model, the long-term safety and the precise synergistic effects of the CSE’s components in human subjects remain to be fully elucidated. In particular, as this study did not include groups treated with pure CBD or other individual cannabinoids, it was difficult to precisely distinguish whether the observed therapeutic effects resulted from CBD alone or from the synergistic interactions between various components in the extract. Therefore, further comparative studies using isolated compounds are necessary to clarify these interactions. Additionally, the CSE preparation process may result in the loss of volatile compounds, such as terpenoids. These constituents are recognized for their essential role in the synergistic effect and the biological activity of *C. sativa* [31]. However, our current study focused on the profile and effects of non-volatile cannabinoids. Therefore, further research is required to analyze these volatile components and elucidate their potential impact on AD. Furthermore, subsequent clinical trials are required to optimize standardized dosages and to confirm the translatability of these findings to human AD patients.

## 5. Conclusions

In conclusion, this study provides a comprehensive scientific basis for the use of CSE by demonstrating its multi-target modulation of inflammatory pathways, specifically the MAPK/NLRP3 and JAK1/STAT6 pathways. These results indicate that CSE is a promising candidate for the management of AD. Despite the recognized limitations, such as the need for comparative studies with isolated compounds, our findings suggest that CSE possesses substantial potential as a valuable nutraceutical and cosmeceutical resource. Ultimately, CSE represents a high-value material with significant prospects for both therapeutic and industrial applications.

## Figures and Tables

**Table 1:** Primer list for qPCR.

Gene		Primer Sequence
*Il-4*	Forward:	5′-ATCATCGGCATTTTGAACGAGGTC-3′
	Reverse:	5′-ACCTTGGAAGCCCTACAGACGA-3′
*Il-13*	Forward:	5′-GCAACGGCAGCATGGTATGGAG-3′
	Reverse:	5′-TGGTATAGGGGAGGCTGGAGAC-3′
*Ccl5*	Forward:	5′-ATATGGCTCGGACACCACTC-3′
	Reverse:	5′-TCTTCTCTGGGTTGGCACACA-3′
*Ccl17*	Forward:	5′-CGAGAGTGCTGCCTGGATTACT-3′
	Reverse:	5′-GGTCTGCACAGATGAGCTTGCC-3′
*Ccl22*	Forward:	5′-TCTGATGCAGGTCCCTATGGT-3′
	Reverse:	5′-TTATGGAGTAGCTTCTTCAC-3′
*Cxcl10*	Forward:	5′-CTGAGTGGGACTCAAGGGAT-3′
	Reverse:	5′-TCGTGGCAATGATCTCAACAC-3′
*Gapdh*	Forward:	5′-CATCACTGCCACCCAGAAGACTG-3′
	Reverse:	5′-ATGCCAGTGAGCTTCCCGTTCAG-3′

**Table 2:** Cannabinoid profile and quantitative analysis of CSE.

Cannabinoid	Content (mg/g)	R.T. (min)	M.W. (g/mol)
CBDV	2.87 ± 0.09	2.66	286.41
CBDVA	1.57 ± 0.12	3.40	330.42
CBD	16.39 ± 0.16	4.61	314.46
CBG	N.D.	4.85	316.48
THCV	N.D.	5.14	286.41
CBDA	261.79 ± 0.6	5.44	358.47
CBGA	5.68 ± 0.18	6.76	360.49
CBN	N.D.	7.41	310.43
Δ^9^-THC	3.97 ± 0.03	8.37	314.46
THCVA	N.D.	8.66	330.42
Δ^8^-THC	N.D.	8.73	314.46
CBL	N.D.	8.91	314.46
CBC	1.27 ± 0.21	9.71	314.46
CBNA	N.D.	10.00	354.44
THCA	6.51 ± 0.1	10.82	358.47

Abbreviations: CBDV, cannabidivarin; CBDVA, cannabidivarinic acid; CBD, cannabidiol; CBG, cannabigerol; THCV, tetrahydrocannabivarin; CBDA, cannabidiolic acid; CBGA, cannabigerolic acid; CBN, cannabinol; Δ^9^-THC, delta-9-tetrahydrocannabinol; THCVA, tetrahydrocannabivarinic acid; Δ^8^-THC, delta-8-tetrahydrocannabinol; CBL, cannabicyclol; CBC, cannabichromene; CBNA, cannabinolic acid; THCA, tetrahydrocannabinolic acid; N.D., not detected.

## Data Availability

Data is contained within the article.
